# Prevalence of 22q11.2 microdeletion in 146 patients with cardiac malformation in a referral hospital of North India

**DOI:** 10.1186/1471-2350-11-101

**Published:** 2010-06-23

**Authors:** Ashutosh Halder, Manish Jain, Isha Chaudhary, Madhulika Kabra

**Affiliations:** 1 Department of Reproductive Biology, All India Institute of Medical Sciences, New Delhi, India; 2 Department of Paediatrics (Genetics Unit), All India Institute of Medical Sciences, New Delhi, India

## Abstract

**Background:**

The 22q11.2 microdeletion syndrome is a common condition that is associated with cardiac as well as extra-cardiac manifestations. Its prevalence and manifestations from north India has not been reported. This study was designed to determine the prevalence and ability of clinical criteria to predict 22q11.2 microdeletion.

**Methods:**

A total of 146 cases of cardiac malformation requiring tertiary care at a teaching hospital were prospectively screened for 22q11.2 microdeletion using fluorescence in situ hybridization test. Detailed clinical information was obtained as per guidelines of Tobias, *et al *(1999).

**Results:**

Nine out of 146 patients (6.16%) was found to have 22q11.2 microdeletion. All the positive patients showed the presence of extra-cardiac features of 22q11.2 microdeletion syndrome. None of the cases with isolated cardiac defect were positive for microdeletion.

**Conclusions:**

It seems that 22q11.2 microdeletion syndrome is over-suspected in children with isolated congenital heart defects. Screening for 22q11.2 microdeletion should be considered in those cardiac malformation cases which have extra-cardiac manifestations in the form of facial dysmorphism and hypocalcaemia.

## Background

The 22q11.2 microdeletion syndrome is characterized by hemizygous microdeletion of 22q11.2 region of chromosome 22. It occurs at a frequency of 1 in 4,000 to 6,000 live births [1], and is mostly spontaneous [2]. Mutation of the TBX1 gene, located in 22q11.2 region, has been suggested as a rare cause of the syndrome [3]. The 22q11.2 microdeletion is found in patients with DiGeorge syndrome, Velocardiofacial syndrome and Conotruncal anomaly face syndromes [4]. Typically, the cardiac anomalies involve conotruncus, and include lesions such as tetralogy of Fallot (TOF: pulmonary stenosis, overriding aorta, ventricular septal defect & right ventricular hypertrophy), truncus arteriosus (TA) [4] and double-outlet right ventricle besides interrupted aortic arch and subaortic ventricular septal defect.

Several studies have tried to ascertain the incidence of this condition in general population [1,5,6]; and in patients with heart disease [7-15], psychiatric disease [16-20], neonatal hypocalcaemia [21] and velopharyngeal insufficiency [22]. However, there are very few studies from India. Most Indian studies are case reports [23-25]. There is only one prospective study from western India [26]. The prevalence and clinical presentation of the disease from north India has not been adequately described. This study was aimed to investigate the prevalence of 22q11.2 microdeletion syndrome in cases with congenital cardiac malformation with or without other congenital anomalies (extra cardiac).

## Methods

From August 2006 to March 2010, 146 cases of structural heart defects, with or without extra-cardiac anomalies including dysmorphic features, that required tertiary care management at the All India Institute of Medical Sciences prospectively enrolled into the study. They were referred from various States of north India for tertiary care management. All the patients have undergone cardiac catheterization or operative procedure as required for their condition. A few older patients were recruited from the outpatients. The study was approved by the Institutional Human Ethics Committee.

All patients underwent cardiac, as well as clinical genetics evaluation including echocardiography and CT/catheter angiography. Clinical genetics evaluation was carried out as per guidelines of Tobias, *et al *(1999) [27] (Table 1). The relevant morphological features were recorded.

**Table 1:** shows clinical features that should lead to consideration of FISH analysis for possible 22q11.2 microdeletion (adapted from Tobias, *et al *1999) [25]

Column A	Column B	Column C
*Presence of one of the following*	*Presence of two or more of the following core features*	*Presence of one core feature plus one of these associated features*
Conotruncal cardiac anomaly such as Fallot's tetralogy, interrupted aortic arch, truncus arteriosus or major aorto-pulmonary collateral arteries	Characteristic facial abnormalities viz. broad bulbous nose, square shaped tip of nose, short philtrum, telecanthus, slanting eyes, low set ears, etc	Long slender fingers and hands
Parent of an affected child	Non-conotruncal congenital cardiac defect	Short stature
	Learning difficulties/developmental delay	Hypotonia
	Cleft palate, velopharyngeal insufficiency or swallowing difficulty	Renal abnormalities or Potter sequence
	Hypocalcaemia	Psychiatric (especially bipolar) disorders
	Immunodeficiency or thymic hypoplasia	Family history of congenital cardiac defects

Molecular cytogenetic study was carried out on both interphase and metaphase cells. EDTA, as well as heparinized blood sample was collected from the affected individuals (0.5-1 ml each). Interphase cell suspension was prepared by standard method [24]. Blood nucleated cells were washed in phosphate buffer saline, three times before hypotonic treatment (50 mMol KCL) for 30 minutes and fixation in methanol:acetic acid solution (3:1). Cells were finally re-suspended in 100 ul of fresh fixative. Approximately 20 ul of cell suspension was used to prepare the slide. Metaphase spreads were prepared from phytohaemagglutinin stimulated human peripheral blood lymphocytes using standard cytogenetic technique.

Microdeletion status was determined by FISH using non-commercial DNA probes. Bacterial artificial chromosome (BAC)/Phage artificial chromosome (PAC) clones RP5-882J5 (22q11.2), RP11-22M5 (22q11.22) & CTA-154H4 (22q11.21) spanning approximately 2 mega base (Mb) in length from 22q11.21 through 22q11.23 (microdeletion detection limit of >90% in patients with DiGeorge anomaly) were obtained from European Resource Centre for Molecular Cytogenetics, University of Bari, Italy http://www.biologia.uniba.it/rmc/; courtesy Professor Mariano Rocchi) for the study. The clones were received as bacterial LB agar stab culture, which were sub-cultured on LB agar plate before growing in large amount in LB medium. Probe DNA was extracted using a commercial BAC extraction kit (Sigma, India). All probes were labelled using nick translation method with FITC-12-dUTP (Roche) or TRITC-12-dUTP (Roche) or Cy3 (Amersham, UK). Working concentration of probe DNA was between 100-200 ng/μl.

Slide was washed in acetic acid for 2 min, and dehydrated in 70, 90, 100% ethanol, 3 min each. Nuclei on the slide were digested with pepsin (100 mg/ml) in 0.01 N HCl for 20 min at 37°C, rinsed in double distilled water and followed by PBS, and fixed in 1 % paraformaldehyde in PBS for 10 min at 4°C. Slides were then rinsed in PBS, twice in double distilled water and then dehydrated through ethanol series as before. The hybridization buffer (60% formamide, 2× SSC, 10% dextran sulphate, Sigma, USA) containing labelled probe was applied to the slides under a circular cover slip (11 mm in diameter). The probes and nuclear DNA were denatured together at 76°C for 6-7 min. Hybridization was performed in a dark moist chamber at 37°C for overnight. After hybridization, cover slips were removed and slides were washed with NP40 (0.03% solution) at 72°C for 2-3 min, followed by NP40 (0.01% solution) for 2 min at room temperature. Then slides were dehydrated in ethanol series, as before and mounted in antifade (Vector, USA) with 1 μg/ml 4,6 diaminidino-2- phenylindol (DAPI; Sigma, USA). The slides were screened under Olympus BX 51 fluorescent microscope with 100 watt mercury bulb using 100× plan-apochromatic objective and single band pass filter for DAPI, FITC and TRITC (Olympus Japan). FISH image was captured through spectral imaging system. A total of at least 1000 interphase nuclei and at least 10 metaphase nuclei were scored from each case. Presence of two signals in 100% metaphases and 90% interphase cells was considered as normal whereas demonstration of one signal in 100% metaphases or 85-90% interphase cells was considered as microdeletion positive. When presence of both one and two signals in interphase (15% deleted) as well as in metaphase (10% deleted) cells was observed, the case was considered to have mosaicism.

## Results

**Figure 1:**
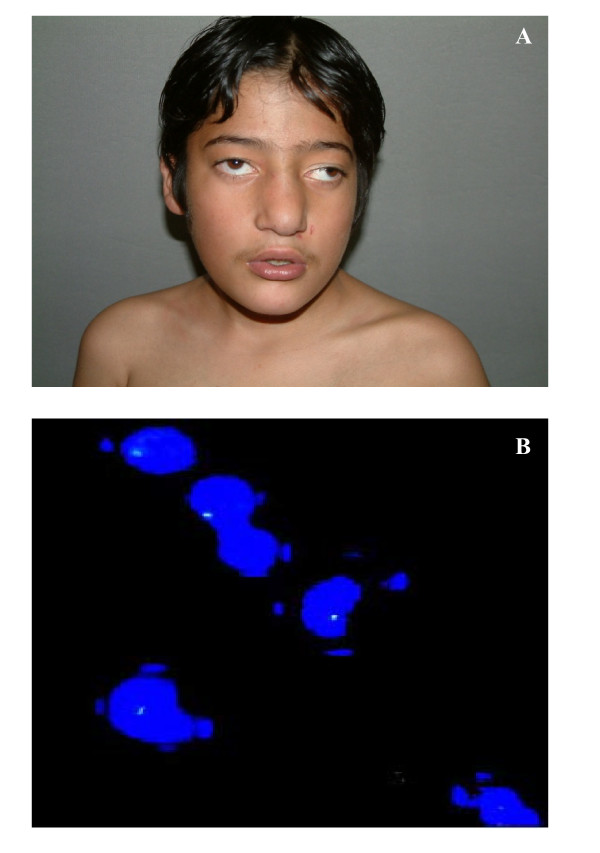
**Non-mosaic 22q11.2 microdeletion case**. (A) Front view of face is showing broad nose, square shaped tip of nose, small philtrum, hypertelorism, telecanthus, squint and low set ears. (B) FISH image is showing one signal, indicating hemizygous 22q11.2 microdeletion.

**Figure 2:**
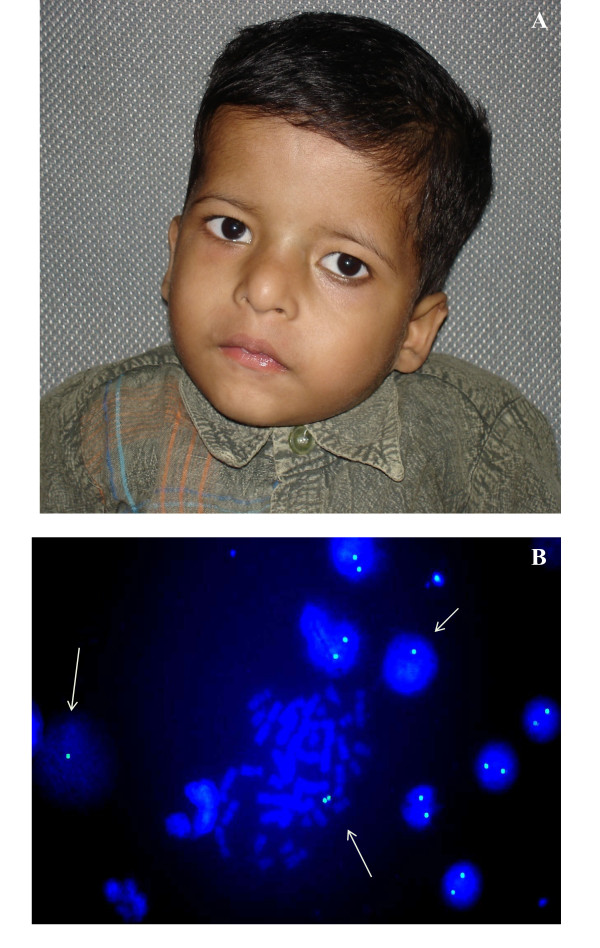
**Mosaic 22q11.2 microdeletion case**. (A) Front view of face is showing broad nose, square shaped tip of nose, small philtrum, hypertelorism, telecanthus, squint and low set ears. (B) FISH image is showing one signal (arrow) as well as two signals, indicating mosaicism for 22q11.2 microdeletion.

A total of 146 consecutive cases with congenital structural heart defect requiring surgical treatment were studied by FISH. The mean age of the patients was 7.7 years, range 5 days to 29 years. There were 97 males and 49 females. Among 146 cases seventy six patients had conotruncal heart defect and 70 had non-conotruncal heart defect (Table 2). Typical clinical features of 22q11.2 microdeletion syndrome were seen in 87 cases. Isolated heart defect was seen in 59 cases. Out of 146 patients, nine patients had hemizygous 22q11.2 microdeletion (6.16%); 6 in non-mosaic form and 3 in mosaic form (Tables 2 and 3; Figure 1 &2). One mosaic patient had trigonocephaly due to craniosynostosis of the metopic suture. No patient with isolated cardiac malformation was found to have a 22q11.2 microdeletion. All patients with microdeletion were detected by RP5-882J5 (22q11.2) probe. Other two probes i.e., RP11-22M5 (22q11.22) & CTA-154H4 (22q11.21) were negative for hemizygous microdeletion. All karyotypes were normal with conventional cytogenetic analysis.

**Table 2:** shows details of structural cardiac malformation in relation to extra-cardiac malformations

Defects	With extra-cardiac malformation	Without extra-cardiac malformation (isolated heart defect)	Total
***Conotruncal Anomalies***	***57***	***19***	**76**
TOF	49	18	67
Non TOF	08	01	09
***Non-Conotruncal Anomalies***	***30***	***40***	**70**
VSD	03	15	18
ASD	00	07	07
Multiple anomalies (2 or more combinations of pulmonary stenosis, VSD, ASD, AVSD, coarctation of aorta, PDA, etc)	27	18	45
**Total**	**87**	**59**	**146**

Conotruncal Anomalies: tetralogy of Fallot, truncus arteriosus communis, double-outlet right ventricle, persistent truncus arteriosus

TOF: Pulmonary stenosis, overriding aorta, ventricular septal defect, right ventricular hypertrophy

Additional findings: stenosis of the left pulmonary artery, bicuspid pulmonary valve, right-sided aortic arch, a foramen ovale, or atrial septal defect, an atrioventricular septal defect

**Table 3:** shows clinical manifestations of patients with 22q11.2 microdeletion (n = 9)

Case	Age	Gender	Religion	Cardiac Abnormality	Extra cardiac Abnormality	Interphase FISH Result *RP5-882J5 (22q11.2) deleted
1	96 months	Female	Muslim	TOF	FD, BD, LD/DD	93% interphase cells with hemizygous microdeletion
2	14 months	Male	Hindu	TOF	FD, HT, LD/DD	98.5% interphase cells with hemizygous microdeletion
3	33 months	Male	Hindu	TOF	FD, HT, SS, LD/DD	Mosaic; 8% normal interphase cells (metaphase: 2 without & 18 with hemizygous microdeletion)
4	18 months	Male	Hindu	TOF	FD, CS, LD/DD	Mosaic; 15% interphase cells with hemizygous microdeletion (metaphase: 1 with & 9 without hemizygous microdeletion)
5	75 days	Male	Hindu	TOF	FD, VPI, DD, HC	96% interphase cells with hemizygous microdeletion
6	55 days	Female	Hindu	TOF	FD, HC	97.5% interphase cells with hemizygous microdeletion
7	60 days	Male	Hindu	TOF	FD, HC	94% interphase cells with hemizygous microdeletion
8	36 months	Female	Hindu	TOF	FD, LD/DD	95% interphase cells with hemizygous microdeletion
9	10 months	Male	Hindu	TOF	FD, DD	Mosaic; 30% interphase cells with hemizygous microdeletion (metaphase: 3 with & 8 without hemizygous microdeletion)

TOF = tetralogy of Fallot; FD = facial dysmorphism; LD/DD = learning difficulties/developmental delay; BD = behavioural disorder; HT = hypotonia; SS = short stature; CS = craniostenosis (metopic suture); HC = hypocalcaemia; VPI = velopharyngeal insufficiency

*Other 2 probes i.e., RP11-22M5 (22q11.22) & CTA-154H4 (22q11.21) were found dizygous (i.e., not deleted)

## Discussion

The association of conotruncal cardiac defect with hemizygosity for the region of 22q11.2 is taken as an evidence for genetic basis of congenital heart defects [28,29]. This syndrome shows wide variability in penetrance and expressivity, hence cases differs each other. As molecular probes for this region are now readily available, the diagnosis of 22q11.2 microdeletion is routinely performed [30,31] despite phenotypic variability.

The cases included in the present study are not representative of the general population as they were specifically referred for tertiary care at our hospital; hence, more likely to be severe cases. In this prospective study, we have found that 6.16% of the structural congenital heart defect patients were positive for 22q11.2 microdeletion (hemizygosity with RP5-882J5 clone). All microdeletion positive cases had characteristic extra-cardiac manifestations besides cardiac defect as tetralogy of Fallot (Table 3). This frequency is towards the lower side of other similar reports (TOF 6% to 21%, PA/VSD 32% to 48%) [32,33]. Although, another report from western India has also found a frequency of 5.7% (with commercially available probes), but in contrast to this report we did not find any 22q11.2 microdeletion in isolated ASD (nil vs 6.6%) or VSD cases (nil vs 12%) [26] (Table 2). A study by Jiang et al (2005) has found 2 of 4 isolated Tetralogy of Fallot and 1 of 5 isolated VSD patients to be positive for 22q11.2 microdeletion [34]. Similarly, Gioli-Pereira et al (2008) have found 22q11.2 microdeletion in 8 of 123 patients (6.5%) with isolated congenital heart defects [35]. This could be due to non recognition of other associated cardiac anomalies (may be due to incomplete investigation) and extra cardiac features (may be overlooked due to severity of the cardiac disease). Such discrepancies can be minimized by adhering to guidelines for clinical diagnosis laid down by Tobias et al. (1999) [27] (Table 1). Guidelines suggest that FISH analysis should be performed on those patients who meet at least one of the criteria in column A. Any patient with a conotruncal cardiac anomaly, even in isolation, deserves to be investigated for presence of 22q11.2 microdeletion, because microdeletion is frequently associated (~50%). Alternatively, history or presence of two features in column B, or one feature in column B and an additional feature in column C regarded as sufficient to merit FISH investigation. These guidelines were proposed with the aim of achieving high sensitivity for FISH analysis. Our experience, through this study, does not support FISH analysis on isolated cardiac malformations. More criteria need to be incorporated for better pick up of microdeletion positive cases. Guidelines for 22q11.2 microdeletion screening have also been reported by Digilio et al (1999) [36]. They favor 22q11 microdeletion testing only in patients with associated classic or subtle clinical anomalies falling within the phenotypic spectrum of 22q11 microdeletion, and in those presenting with distinct anatomic conotruncal defect subtypes. Our study supports these guidelines.

This syndrome is inherited from parents in 5-10% of cases with 50% risk for transmission. Hence, it is recommended (also by Tobias et al. 1999) to consider FISH analysis for possible 22q11.2 microdeletion to parents of all affected child. It seems also reasonable to offer prenatal diagnosis where required.

## Conclusion

It is concluded that 22q11.2 microdeletion syndrome is over-suspected in children with isolated congenital heart defects. We recommend routine FISH testing in cases with conotruncal congenital heart disease associated with extra cardiac anomalies (e.g., dysmorphic features or hypocalcaemia).

## Consent

Written informed consent was obtained from the parents of patients for publication of cases and accompanying images. A copy of the written consent is available for review by the Editor-in-Chief of this journal.

## Competing interests

The authors declare that they have no competing interests.

## Authors' contributions

AH formulated activity plan, checked & interpreted results, besides being the Principal investigator of the project funded by Indian Council of Medical Research, India. He also reviewed clinical findings, prepared manuscript and responded to the queries of reviewers. He is the guarantor of the manuscript. MJ carried out all FISH related activity under guidance of AH, besides working as Research fellow for the project under AH. IC also carried out FISH related activity under guidance of AH. MK was involved in clinical management of the cases. All authors read and approved final manuscript.

## Pre-publication history

The pre-publication history for this paper can be accessed here:

http://www.biomedcentral.com/1471-2350/11/101/prepub
